# Instrumental Food-to-Bolus Transformation of Commercial Toddler-Oriented Finger Foods: An Exploratory Comparison

**DOI:** 10.3390/foods15213771

**Published:** 2026-10-22

**Authors:** Aunchalee Aussanasuwannakul

**Affiliations:** Department of Food Chemistry and Physics, Institute of Food Research and Product Development, Kasetsart University, Bangkok 10903, Thailand; aunchalee.a@ku.th or ifrala@ku.ac.th; Tel.: +66-029428629

**Keywords:** food oral processing, complementary feeding, food texture, oral disintegration, bolus formation, texture analysis, rheology, tribology, acoustic emission, viscoelasticity

## Abstract

Commercial complementary finger foods vary widely in structure, yet conventional product categories provide limited information on how they transform during oral processing. This exploratory study characterized seven commercial toddler-oriented snacks using a multi-stage instrumental approach linking mechanical–acoustic fracture with saliva-mediated disintegration and hydrated-bolus mechanics. Products were assigned to provisional descriptive matrix categories: protein-based cellular, expanded starch-based cellular, and dense biscuit matrices. Three purposively selected exemplars—yogurt melts (P2), mini crackers (P4), and milk biscuits (P5)—were further evaluated by artificial saliva-mediated oral disintegration, dynamic instrumental mastication (DIM), rheology, and tribology. Dry-state hardness varied more than six-fold among the seven products (531.6–3426.3 g), with only partial correspondence between provisional matrix category and fracture profile. P2 showed rapid structural disruption, with largest core proportion decreasing from 98.9% to 18.5% by 30 s; P4 showed progressive disintegration and required the highest saliva-to-sample ratio for cohesive-bolus formation (2.5:1); and P5 retained coarse fragments and formed the bolus with the highest storage modulus (*G*′ = 616.6 Pa at 1 rad s^−1^). Despite rapid disintegration, P2 showed the highest initial DIM force (9.30 N) and highest integrated friction (nAUC_log _*_v_* = 0.154). These product-specific findings suggest associations between initial product characteristics and subsequent instrumental responses, with rankings varying across measurements. They do not establish class-wide pathways or physiological oral-processing performance.

## 1. Introduction

Complementary feeding is the period during which foods and beverages are introduced in addition to milk as milk alone becomes insufficient to meet nutritional requirements, generally beginning at about 6 months and continuing through 23 months of age [1]. During this period, food consistency and variety should progress in accordance with children’s developmental abilities, supporting the transition toward increasingly complex complementary foods [1]. Importantly, there is no single precise chronological age at which all infants acquire readiness for different complementary-food textures, because developmental readiness varies among individuals and differs according to the feeding task involved [2]. Empirical observations likewise show rapid changes in the textures consumed between 8 and 12 months of age, reflecting the continuing development of feeding skills over a relatively short developmental interval [3]. Food texture is therefore not merely a product-quality attribute during complementary feeding but is closely linked with the development and expression of food oral-processing skills and eating behavior [4].

Commercial foods intended for infants and young children encompass a broad range of finger-food and snack formats, including puffs, rice cakes, crackers, biscuits, wafers, rusks, and related cereal-based products [5]. Codex similarly recognizes rusks and biscuits as processed cereal-based complementary foods and specifies that products prepared according to label instructions should have a texture appropriate for their intended age [6]. Commercial complementary foods are therefore encountered across diverse product categories and feeding stages, yet conventional category names or age/stage positioning do not provide standardized quantitative information on how the products mechanically break down and transform during eating. This creates an important distinction between commercially useful product descriptors and the underlying physical properties that govern oral handling.

Food oral processing describes the progressive transformation of food in the mouth through mechanical breakdown, interaction with saliva, structural reorganization, and formation of a bolus suitable for oral transport and subsequent swallowing [7]. Current food oral-processing frameworks emphasize that this transformation proceeds through interacting processes of comminution, hydration and dilution, particle agglomeration, and bolus formation, during which the physical properties of the material progressively depart from those of the intact food [8,9,10]. Food texture can strongly modify this process: differences in initial structure influence oral-processing behavior, saliva incorporation, particle-size characteristics, and the properties of the resulting bolus [11]. Initial mechanical hardness also influences the extent of comminution, with harder foods producing different particle-size distributions at the point at which adults judge the bolus ready for swallowing [12]. For brittle low-moisture foods such as biscuits, mastication produces substantial structural transformation as particles are fragmented, hydrated by saliva, and progressively agglomerated into a cohesive bolus [13]. Thus, oral behavior cannot be inferred from dry-state hardness or fracture properties alone, because the material present late in oral processing may differ fundamentally from the original food structure. Moreover, the properties of the resulting food–saliva system cannot be described by bulk mechanical or rheological behavior alone, because lubrication at interacting oral surfaces represents a complementary physical dimension of oral processing and may depend on material–surface interactions as well as bulk properties [14,15].

This distinction is particularly relevant for dry complementary finger snacks, which encompass markedly different physical architectures. Expanded cellular snacks, for example, can undergo pronounced changes in mechanical and acoustic properties following exposure to artificial saliva, and the magnitude of these changes varies among products and testing conditions [16]. Freeze-dried dairy matrices may likewise differ substantially in porosity and microstructural organization depending on composition and formulation, indicating that apparently similar porous products need not share identical hydration and structural-collapse behavior [17]. At later stages of oral processing, lubrication behavior may likewise depend on microstructural characteristics and interactions between food constituents and saliva [18]. More generally, food oral-processing research emphasizes that foods intended for specific populations should be designed and evaluated through approaches that capture relevant transformations from initial structure through later oral-processing stages, rather than relying on a single instrumental endpoint [19]. Contemporary instrumental approaches likewise provide complementary information across mechanical, rheological, and tribological domains, supporting multi-method characterization when food texture cannot be adequately represented by a single physical measurement [20,21,22]. These approaches interrogate different aspects of food breakdown, deformation, bulk flow, and interfacial lubrication and are therefore complementary rather than interchangeable when characterizing oral-processing behavior. Recent work on crispy extruded snacks further demonstrates the value of integrating mechanical–acoustic fracture characteristics with later-stage tribological behavior to identify distinct texture phenotypes that cannot be resolved from force-based measurements alone [23].

Despite these advances, an important question remains insufficiently resolved: whether differences among dry commercial complementary finger foods are associated with contrasting responses across fracture, saliva-mediated disintegration, cohesive-bolus formation, whole-bolus deformation, bulk flow, and interfacial lubrication. Contemporary food oral-processing research recognizes that food structure and texture evolve during breakdown and bolus formation rather than remaining fixed properties of the intact food [24,25]. However, measurements of these transformations interrogate different stages or physical phenomena, and their relationships within a common characterization framework remain less clearly resolved. Combining these measurements may provide a more comprehensive description of food-to-bolus transformation than conventional product names or any single instrumental endpoint. In the present study, commercial toddler-oriented snacks were organized into provisional descriptive matrix categories encompassing protein-based cellular matrices, expanded starch-based cellular matrices, and dense biscuit matrices. These categories guided product selection and interpretation rather than representing quantitatively validated structural classes.

Accordingly, this exploratory study aimed to characterize differences among seven commercial toddler-oriented snacks using mechanical–acoustic fracture measurements and to examine three purposively selected exemplars using saliva-mediated disintegration, repeated whole-bolus compression, rheology, and tribology. Specifically, the study addressed three questions: (1) how the seven sampled products differed in their mechanical–acoustic fracture characteristics; (2) whether selected products with contrasting fracture profiles exhibited different in vitro disintegration and whole-bolus deformation responses; and (3) how their separately prepared hydrated boluses differed in rheological and tribological properties. We hypothesized that contrasting initial product characteristics would be associated with distinguishable responses across these complementary instrumental measurements.

## 2. Materials and Methods

### 2.1. Commercial Snack Samples

Seven commercially available snack products positioned for infants and young children during the complementary-feeding stage were purchased from retail outlets and online marketplaces in Thailand between June and July 2026. Products were purposively selected to include contrasting formulations and apparent physical structures. This exploratory comparison was not designed to represent the commercial population of complementary finger-food snacks. For consistency throughout the study, products were coded P1–P7 and referred to using simplified product descriptions: P1, mini meringue; P2, yogurt melts; P3, rice rusk; P4, mini cracker; P5, milk biscuit; P6, rice cracker; and P7, button cracker.

Commercial identities, manufacturer information, ingredients, package sizes, age recommendations, manufacturing and best-before dates, manufacturer-declared claims, and measured proximate compositions are provided in Supplementary Table S1. Manufacturing-lot identity was not verified; dates identify the sampled material but do not establish lot independence. Age recommendations were treated as commercial descriptors rather than experimental classification criteria.

**Figure 1:**
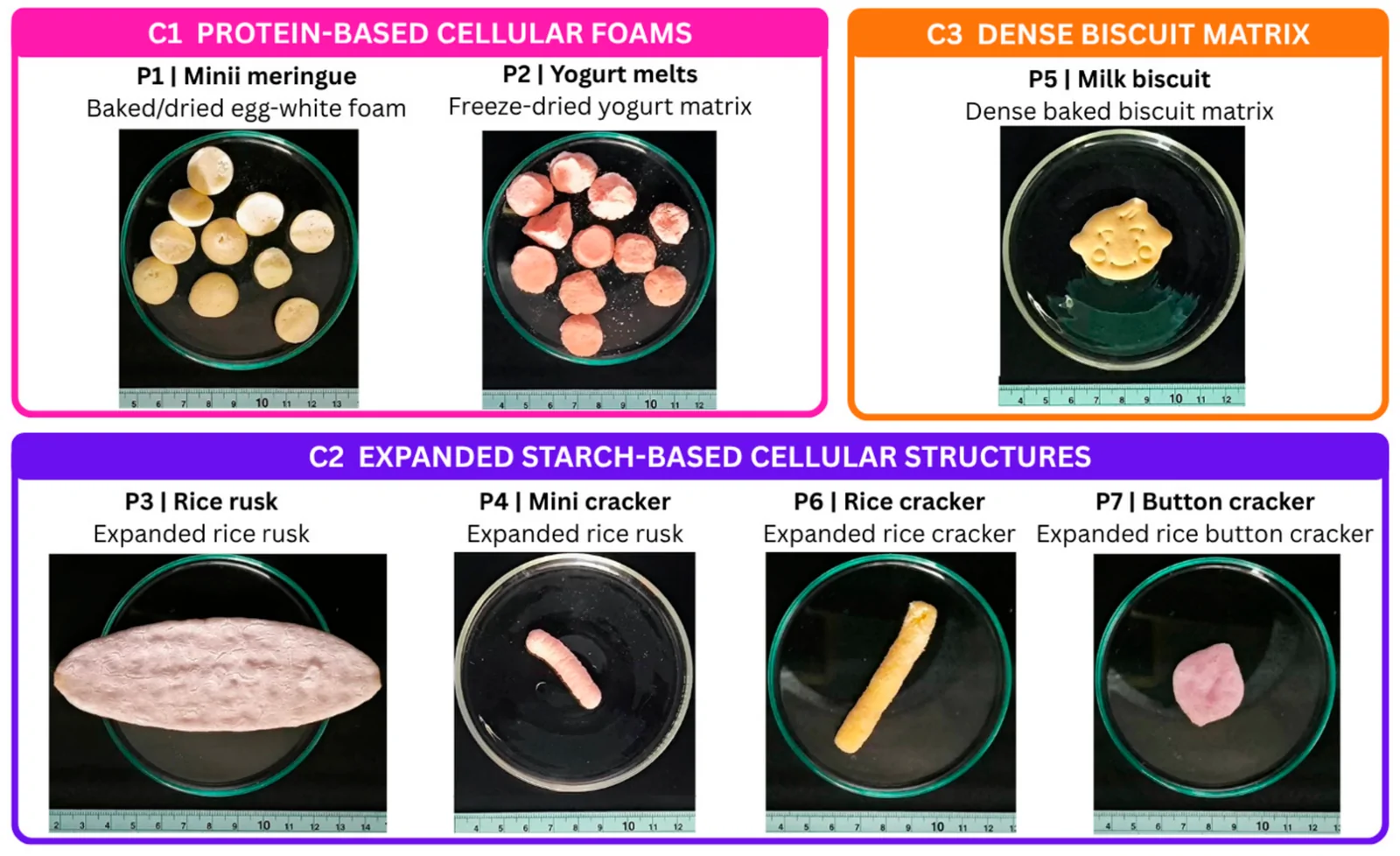
Seven commercial toddler-oriented snack products arranged according to provisional descriptive matrix category: C1, protein-based cellular; C2, expanded starch-based cellular; C3, dense biscuit. Scale rulers indicate centimeters.

Products were assigned to provisional descriptive matrix categories using manufacturer-declared ingredients and qualitative inspection of visible characteristics: C1, protein-based cellular (P1 and P2); C2, expanded starch-based cellular (P3, P4, P6, and P7); and C3, dense biscuit (P5). These categories organized product-specific comparisons; porosity, density, and cellular microstructure were not quantitatively measured to validate structural classes. Representative photographs are shown in Figure 1.

Products were maintained in their original unopened packaging under ambient laboratory conditions (25 ± 2 °C) until analysis and were opened immediately before testing to minimize environmental moisture uptake. All seven products underwent proximate composition and mechanical–acoustic fracture characterization. P2, P4, and P5 were selected for artificial saliva-mediated disintegration, DIM, rheology, and tribology using the purposive selection procedure described in Section 2.2.2.

#### Proximate Composition

Proximate composition of the seven commercial snack products was determined in triplicate using AOAC International methods. Moisture content was determined by drying approximately 2.0 g of sample at 130 °C for 1 h (Method 925.10). Ash, total fat, and crude protein were determined using Methods 942.05, 996.01, and 2001.11, respectively. Crude protein was determined by the Kjeldahl method using a nitrogen-to-protein conversion factor of 6.25. Total carbohydrate content was calculated by difference:

Carbohydrates (%) = 100 − (moisture% + ash% + crude protein% + total fat%)

Results were expressed as percentages on an as-is basis and are provided in Supplementary Table S1.

### 2.2. Mechanical–Acoustic Fracture Characterization and Product Selection

#### 2.2.1. Acoustic-Mechanical Measurement

Mechanical fracture behavior and associated airborne acoustic emissions were measured simultaneously using a texture analyzer (TA-XTplus^®^, Texture Technologies Corp., Scarsdale, NY, USA) equipped with a 50-kg load cell and controlled by Texture Exponent software (v6.2.6.0; Stable Micro Systems Ltd., Godalming, UK), with an integrated Acoustic Envelope Detector (AED; A/RAED) fitted with a ½-in pre-polarized condenser microphone, following the general approach of [23]. Individual snack pieces were subjected to three-point bending (3PB; Application Study REF: PTZ1/3PB) by positioning each sample centrally across two lower supports and applying the central blade. Tests used Compression mode with Return to Start, pre-test and test speeds of 1.0 mm/s, a post-test speed of 10.0 mm/s, a programmed travel distance of 10 mm after the 5-g automatic force trigger, and break detection disabled. Force and acoustic signals were acquired synchronously at 500 points/s. The AED microphone was positioned at approximately 45° and 1 cm from the fracture zone, with the gain set at 24 dB and the envelope corner frequency at 3.125 kHz. Four to eight separate snack pieces were tested once per product (*n* = 4–8); each piece constituted the specimen-level experimental unit.

Mechanical parameters were extracted using the PTZ1/3PB macro: hardness (g) was defined as the maximum positive force in the recorded curve, and fracturability (mm) as the recorded distance coordinate at that force maximum. Acoustic parameters were extracted from the synchronized AED signal using the Texture Exponent Basic Acoustic Macro. Count_Peaks was calculated as the number of positive acoustic peaks exceeding a threshold of 50 raw acoustic units within the selected measurement region. Jagged_Smooth_Ratio (%) was calculated as 100 × the linear distance of the original acoustic trace divided by that of a trace smoothed by averaging every 10 data points. Higher values indicated greater acoustic-trace irregularity.

Product differences in each descriptor were evaluated using the procedures in Section 2.6. Hardness was analyzed using Welch’s ANOVA because variances were unequal; no hardness pairwise comparisons were reported. *η*^2^ was calculated as the conventional between-product sum of squares divided by the total sum of squares and reported descriptively.

#### 2.2.2. Principal Component Analysis (PCA) and Product Selection

Exploratory PCA was performed on the product-level means of hardness, fracturability, Count_Peaks, and Jagged_Smooth_Ratio, with one observation per product (*n* = 7). Variables were standardized, and PCA used the Pearson correlation matrix. Product identity labeled observations; provisional matrix category was supplementary and did not contribute to component calculation. The first two components were displayed in a distance biplot, with explained variance, loadings, contributions, and product scores examined.

P2, P4, and P5 were purposively selected to include one exemplar per provisional category while providing contrasting measured fracture profiles. PCA supported exploratory visualization and selection; it did not validate categories, establish stable population-level grouping, or make the selected products statistically representative.

### 2.3. Artificial Saliva-Mediated Oral Disintegration Assay (ASODA)

#### 2.3.1. Oral Disintegration Test

Artificial saliva was prepared using the formulation reported by Aussanasuwannakul and Kantrong [23]. The solution contained sodium chloride (0.117 g/L), potassium chloride (0.149 g/L), sodium bicarbonate (2.100 g/L), porcine gastric mucin (Type II, Sigma-Aldrich, St. Louis, MO, USA; M2378; 1.000 g/L), and α-amylase from *Bacillus* sp. (Sigma-Aldrich, St. Louis, MO, USA; A6814; 0.050 g/L; nominal activity approximately 4000 U/L). Components were dissolved in LC–MS-grade double-distilled water. The artificial saliva was freshly prepared before each experiment and was equilibrated to 37 °C before disintegration testing. This formulation models selected electrolyte, lubrication, and enzymatic aspects of food–saliva interactions.

Artificial-saliva viscosity and friction were characterized using the rheometer, geometries, and measurement procedures described in Section 2.5, with the following differences. Viscosity measurements were performed at 25 °C, with apparent viscosity extracted at 10 s^−1^. Tribological characterization was performed at 37 °C, with conditioning at 0.01 mm/s for approximately 60 s followed by approximately 5 s at rest. Friction descriptors were calculated as described in Section 2.5. pH, viscosity, and tribological measurements were each performed on three independently prepared saliva samples (*n* = 3).

In vitro disintegration was assessed using an artificial saliva immersion assay combining controlled hydration, agitation, and image analysis [26]. Intact P2, P4, and P5 specimens without visible defects were weighed individually. Five separate specimens were assessed once at each observation time (0, 15, 30, 60, 90, and 120 s; *n* = 5 per product per time point), using destructive sampling. For 15–120-s exposures, each specimen was placed in a separate 45-mL centrifuge tube, and 10 mL of artificial saliva pre-equilibrated to 37 °C was added immediately before agitation. Tubes were maintained at 37 °C in a rotating mixer/incubator (Roto-Therm Mini Plus, Benchmark Scientific, Sayreville, NJ, USA), operating in Mix mode at 30 rpm with combined rotational and oscillatory motion. Timing began upon saliva addition and initiation of agitation. At the designated time, tube contents were gently transferred to a Petri dish and photographed under fixed conditions. For the 0-s condition, 10 mL of artificial saliva was poured over the specimen directly in the Petri dish, which was photographed immediately without agitation.

#### 2.3.2. Visual Assessment and Image Acquisition

Disintegration was assessed visually using a six-point ordinal scale ranging from 0 (intact; no visible change) to 5 (complete disintegration; only small fragments or slurry remaining). Intermediate scores described progressive stages of surface softening, edge erosion, partial collapse, and extensive fragmentation. Visual disintegration scores were used descriptively to support interpretation of the photographic disintegration sequences and were not subjected to inferential statistical analysis.

Images were acquired using a smartphone camera (Xiaomi 13T, Xiaomi Communications Co., Ltd., Beijing, China) mounted at a fixed height with 4× zoom, constant illumination, and a black background. A 90-mm internal-diameter Petri dish was centered in the field of view and used for spatial calibration. Imaging distance and resolution were held constant.

#### 2.3.3. Image Analysis and Derived Parameters

Images were analyzed using Fiji/ImageJ (ImageJ version 1.54p; National Institutes of Health, Bethesda, MD, USA) following a standardized workflow developed for this study. The interior region of the Petri dish was isolated using the Oval Selection tool, and pixels outside the dish were removed. Images were spatially calibrated against the known internal Petri dish diameter (90 mm), converted to 8-bit grayscale, and thresholded at 97–255 to separate food particles from the background. Particle analysis was performed using a minimum particle area of 0.5 mm^2^ and a circularity range of 0.00–1.00, with particles touching the image boundary excluded.

After visual inspection and exclusion of artefacts, the largest detected particle was designated operationally as the ‘largest core.’ Total particle count and projected area were obtained from Fiji/ImageJ, and the following descriptors were calculated:

$$\mathrm{Largest}\mathrm{core}\mathrm{proportion}\left(\%\right)=\frac{\mathrm{largest}\mathrm{core}\mathrm{area}}{\mathrm{total}\mathrm{particle}\mathrm{area}}\times 100$$

Fragment count = total particle count − 1

Fragment total area (mm^2^) = total particle area − largest core area

Largest core proportion describes relative detected projected area, rather than retained food mass or verified structural retention. Fragment count and area describe the other detected particles. These descriptors cannot distinguish fragmentation, dispersion, dissolution, aggregation, or loss below detection criteria. Each specimen image yielded one observation per descriptor; individual particles were not experimental replicates.

Image-derived measurements were processed in Microsoft Excel for Mac (version 16.114; Microsoft Corporation, Redmond, WA, USA). The effect of disintegration time was evaluated separately for each product. Largest core proportion was analyzed by one-way analysis of variance (ANOVA), with time as a categorical fixed factor. Fragment count was log_10_(fragment count + 1)-transformed before ANOVA to accommodate positive skewness and zero values. Fragment total area, which did not adequately satisfy parametric assumptions, was analyzed using the Kruskal–Wallis test with Monte Carlo estimation (10,000 simulations), followed by Dunn’s pairwise comparisons with Bonferroni adjustment when appropriate. General procedures for assumption testing, multiple comparisons, software, and statistical significance are described in Section 2.6.

### 2.4. Dynamic Instrumental Mastication (DIM)

#### 2.4.1. Preparation of Hydrated Boluses

Hydrated boluses of the selected exemplars P2, P4, and P5 were prepared for repeated-compression testing. Samples were mixed with artificial saliva at 37 °C to an operational cohesive-bolus endpoint without visible dry particles or phase separation. Saliva-to-sample ratios were 1.4:1, 2.5:1, and 1.3:1 (*w*/*w*) for P2, P4, and P5, respectively. This endpoint was not validated as a physiological swallowing endpoint. Whole boluses were transferred immediately to the rheometer.

#### 2.4.2. Dynamic Instrumental Mastication Test and Derived Parameters

Dynamic instrumental mastication was adapted from the instrumental mastication assay of [27] and performed using a rheometer (MCR 302, Anton Paar GmbH, Graz, Austria) equipped with a 50-mm parallel-plate geometry maintained at 37 °C. The hydrated bolus was placed centrally on the lower plate and subjected to ten consecutive compression–fixed-gap–decompression cycles to characterize its response to repeated controlled deformation. The protocol was inspired by tongue–palate compression but was not calibrated to toddler oral movements or forces.

During the first cycle, the upper plate moved from an initial gap of 20 mm to a compression gap of 1 mm at a constant vertical speed of 17.8 mm/s. At 1 mm, a shear rate of 10 s^−1^ was applied for 2 s. The plate was then raised from 1 to 3 mm at 17.8 mm/s. At the 3-mm gap, a shear rate of 10 s^−1^ was maintained, comprising an initial 2-s stage followed by a 60-s fixed-gap stage. Cycles 2–10 subsequently compressed the previously deformed bolus from 3 to 1 mm at 17.8 mm/s, followed by the same 2-s fixed-gap stage at 1 mm with a shear rate of 10 s^−1^ and decompression from 1 to 3 mm. Force–time data were continuously recorded using RheoCompass™ software (Version 1.30, Anton Paar GmbH), and the maximum positive normal force during each compression cycle was extracted as the cycle-specific compression force (F1–F10).

Peak positive normal forces were extracted for cycles 1–10. Summary descriptors were initial force (F1), second-to-first-cycle force ratio (F2/F1), and mean peak force over cycles 6–10, termed plateau force.

Three boluses were prepared separately per product (*n* = 3). Each preparation yielded one value per summary descriptor; sequential compression cycles did not increase replication. Product comparisons followed Section 2.6.

### 2.5. Rheological and Tribological Characterization

Artificial boluses of P2, P4, and P5 were prepared using the endpoint-based hydration procedure described in Section 2.4.1. Rheological and tribological properties were characterized using a rotational rheometer (MCR 302, Anton Paar GmbH, Graz, Austria) at 37 °C. Rheological measurements used a 25-mm parallel-plate geometry, whereas tribological measurements used a ball-on-three-pins attachment.

Steady-shear measurements were performed using a logarithmic shear-rate ramp from 0.001 to 10 s^−1^ with 17 measurement points. Oscillatory amplitude sweeps were conducted over a strain range of 0.01–100% at a constant angular frequency of 10 rad s^−1^ using 25 measurement points. Frequency sweeps were subsequently performed from 100 to 0.1 rad s^−1^ at a constant strain of 0.1%, selected from the apparent low-strain plateau observed in the amplitude sweeps, using 16 measurement points. Tribological measurements were conducted under a constant normal force of 1 N following preconditioning at 0.01 mm s^−1^ for 30 s and equilibration for 2.5 s. The friction coefficient (*μ*) was then recorded over a logarithmic sliding-velocity range of 0.01–1000 mm s^−1^ using 61 measurement points to generate Stribeck curves.

For each preparation, the Ostwald–de Waele model, *τ* = *K*$\dot{\gamma }$*^n^* was fitted by linear regression of log_10_(shear stress) against log_10_(shear rate) over 0.1–10 s^−1^. Here, *τ* is shear stress (Pa), $\dot{\gamma }$ is shear rate (s^−1^), *K* is the consistency coefficient (Pa·s*^n^*), and *n* is the flow behavior index. Fit adequacy was assessed using log-scale R^2^ and residuals. *K* and *n* were reported descriptively only where the model was adequate, without inferential comparisons. Apparent viscosity at 1 s^−1^ (*η*_1_) was extracted from the measured curve.

Amplitude sweeps provided storage modulus at 1% strain (*G*′_1%_), the software-derived LVE limit (*γ*_c_), and flow-point stress (*τ*_f_), defined as the shear stress at the crossover between *G*′ and *G*″. Critical strain (*γ*_c_) was determined using the RheoCompass LVE-range analysis as the strain at which *G*′ left a ±3% tolerance band around its initial plateau, with curve smoothing enabled (1% smoothing range). Frequency sweeps provided storage modulus (*G*′), loss modulus (*G*″), and loss tangent (tan *δ* = *G*″/*G*′), with values extracted at 1 rad s^−1^ for product comparison.

Tribological descriptors comprised the boundary friction coefficient (*μ*_B_), defined as the friction coefficient at the lowest sliding velocity (0.01 mm s^−1^); high-speed friction coefficient (*μ*_high_), defined as the friction coefficient at the highest sliding velocity (1000 mm s^−1^); and normalized log-velocity area under the Stribeck curve (nAUC_log _*_v_*), calculated by trapezoidal integration of *μ* against log_10_-transformed sliding velocity and normalized to the total logarithmic velocity range.

Three boluses were prepared separately per product for each measurement protocol (*n* = 3), with each preparation constituting the experimental unit. Curve points were used to derive preparation-level descriptors and did not constitute additional replicates. Descriptors other than *K* and *n* were compared using the procedures in Section 2.6. Flow-point stress was analyzed using the Kruskal–Wallis test with Monte Carlo estimation (10,000 simulations), followed by Dunn’s pairwise comparisons with Holm–Bonferroni adjustment.

### 2.6. Data Processing and Statistical Analysis

Quantitative data are presented as mean ± standard deviation (SD) of the specimens, sample portions, or bolus preparations specified for each method. Replication within the sampled commercial material did not establish independent manufacturing-lot replication. Statistical comparisons concern that material under the tested conditions and do not estimate between-lot variability or establish differences between entire structural categories.

Statistical analyses were performed using XLSTAT (version 2026.1.1.1434, Addinsoft, Paris, France). Residual normality and variance homogeneity were assessed using the Shapiro–Wilk test and median-centered Levene’s test, respectively. Conventional one-way ANOVA was followed by Tukey’s honestly significant difference (HSD) test when the overall test was significant and assumptions were adequately satisfied. Welch’s ANOVA, transformations, and nonparametric procedures were applied as specified in the corresponding analytical subsections. Statistical significance was accepted at *p* < 0.05.

## 3. Results

### 3.1. Mechanical–Acoustic Fracture Characteristics and Product Differentiation

#### 3.1.1. AED-3PB Characteristics

**Figure 2:**
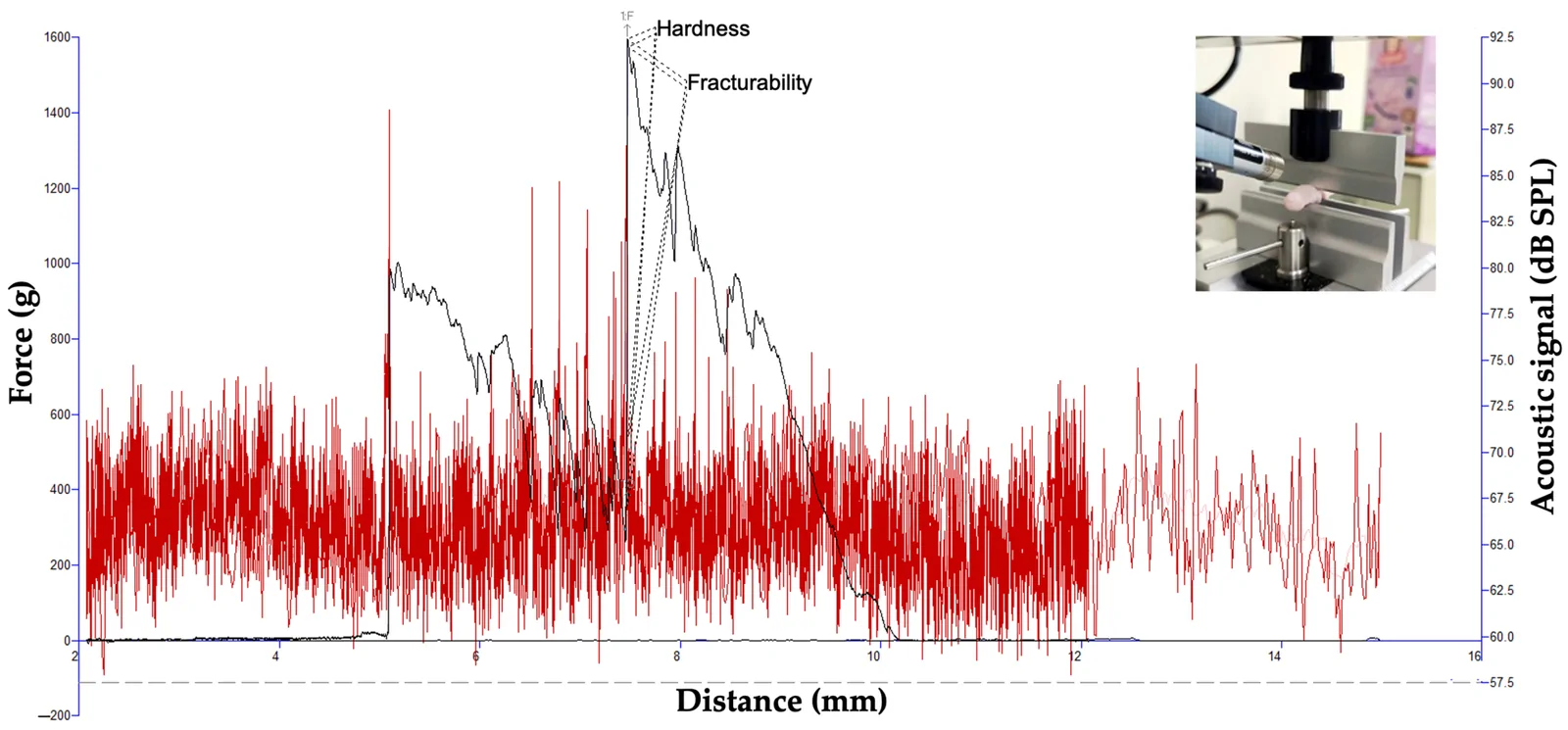
Representative mechanical–acoustic profile obtained during three-point bending (3PB) of a commercial snack, showing the synchronized force–distance and acoustic signals. The black curve represents force (left axis), and the red curve represents the acoustic signal (right axis). Hardness was defined as the maximum positive force in the recorded curve, and fracturability as the recorded distance coordinate at that force maximum. The inset shows the 3PB configuration and microphone positioning used for simultaneous mechanical and acoustic measurements.

Figure 2 illustrates a representative synchronized force–distance and acoustic profile obtained during three-point bending, indicating the force maximum and its corresponding recorded distance coordinate.

The seven sampled commercial products exhibited substantial differences in AED-3PB fracture characteristics (Table 1). Comparisons based on separately tested snack specimens identified significant differences among the sampled products for all four parameters, although the magnitude of differentiation varied among descriptors. Hardness differed significantly among the sampled products. The Shapiro–Wilk test did not reject residual normality (*p* = 0.051), whereas the homoscedasticity test indicated unequal variances (*p* = 0.009). Welch’s ANOVA therefore served as the primary overall test and identified differences among product means, F(6, 11.22) = 75.218, *p* < 0.0001.

Fracturability also differed significantly among products (*p* < 0.0001, *η*^2^ = 0.861). P7 and P3 exhibited the lowest fracture distances (2.63 and 3.62 mm, respectively), whereas P2 exhibited the highest value (7.00 mm). P5 showed an intermediate fracture distance, while P1, P4, and P6 exhibited relatively high and statistically overlapping values.

The acoustic descriptors also differentiated the products. Count_Peaks differed significantly among products (*p* = 0.003), although its smaller effect size (*η*^2^ = 0.461) and extensive overlap among Tukey groups indicated weaker discrimination than the other descriptors. P2 exhibited the highest mean Count_Peaks and differed significantly from P4 and P7. In contrast, Jagged_Smooth_Ratio showed the strongest product effect (*p* < 0.0001, *η*^2^ = 0.873), ranging from 622.0 for P3 to 885.2 for P6. P3 exhibited the lowest value, followed by P7, whereas P2, P5, and P6 exhibited comparatively high values.

#### 3.1.2. Multivariate Differentiation and Product Screening

**Figure 3:**
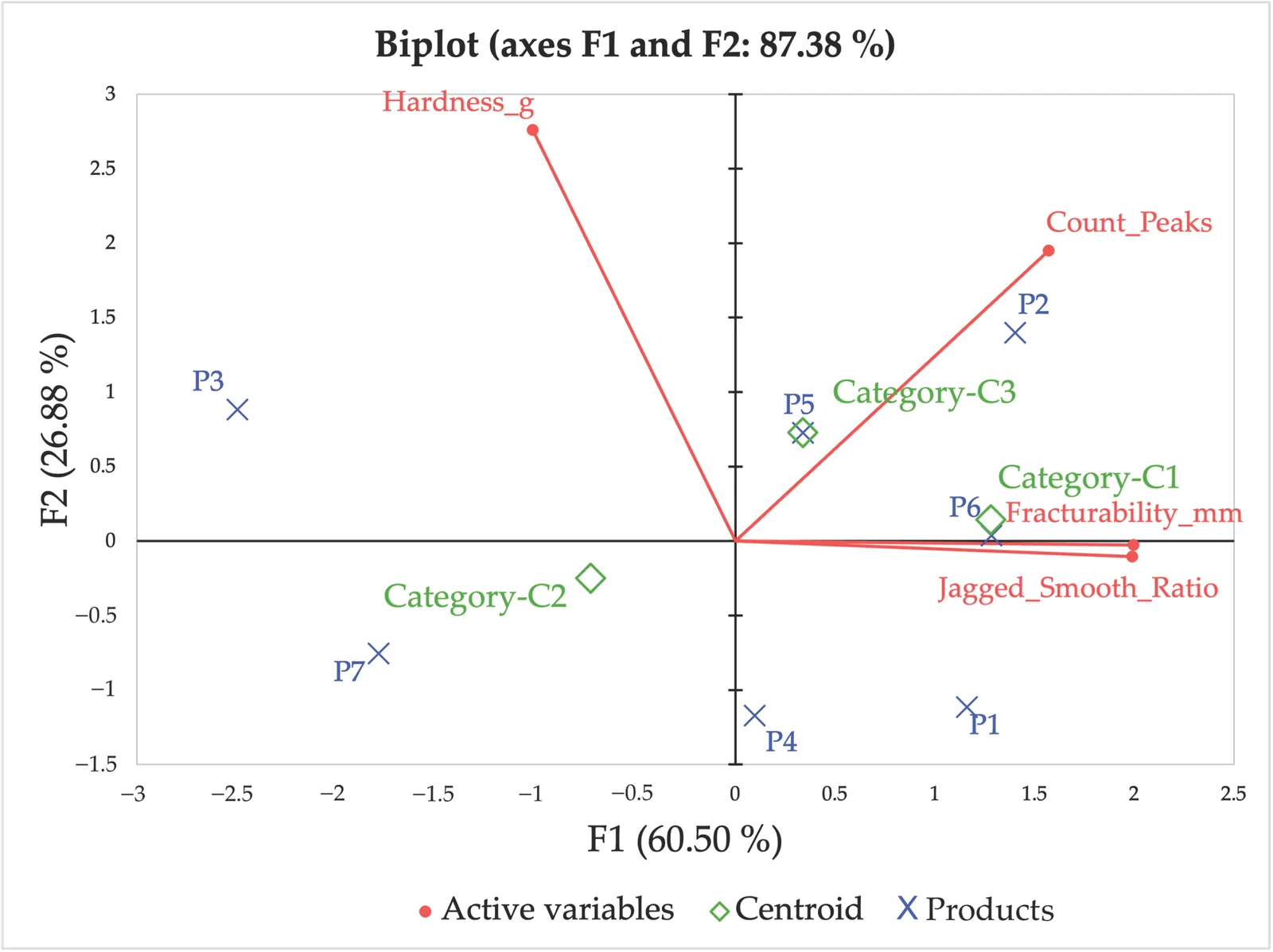
Principal component analysis biplot of product-level mean AED-3PB fracture characteristics of seven commercial toddler-oriented snack products. Each product point represents the mean of separately tested snack specimens, yielding seven product-level observations for PCA (*n* = 7). PC1 and PC2 explained 60.50% and 26.88% of the total variance, respectively. Vectors represent the active variables: hardness, fracturability, Count_Peaks, and Jagged_Smooth_Ratio. Blue crosses represent individual products, red lines with circular endpoints represent active variables, and green unfilled diamonds indicate descriptive category centroids. Matrix category was included as a supplementary qualitative variable and did not contribute to construction of the principal components. The C3 centroid coincides with P5, its sole sampled product; centroids do not establish category separation.

Exploratory PCA visualized differences among the product-level mean AED-3PB profiles of the seven sampled products (Figure 3). The first two principal components jointly explained 87.38% of the total variation, with PC1 and PC2 accounting for 60.50% and 26.88%, respectively. The corresponding variable loadings and contributions are summarized in Table 2.

PC1 was most strongly associated with fracturability and Jagged_Smooth_Ratio, whereas PC2 was dominated by hardness, with Count_Peaks contributing to both axes (Table 2).

The biplot showed contrasting product profiles (Figure 3). P2 and P5 occupied the positive-PC1/positive-PC2 region, whereas P3 combined positive PC2 with negative PC1. P1 and P4 had negative PC2 scores, P7 was negative on both axes, and P6 had a positive PC1 score near the PC2 origin.

The provisional matrix categories showed only partial correspondence with the multivariate distribution. C1 products were positioned toward the positive region of PC1, whereas the C2 products were widely dispersed across the PCA space, indicating variation among the sampled C2 products. C3 was represented solely by P5. These positions describe the seven sampled products and do not establish separation or characteristic fracture profiles of the categories.

P2, P4, and P5 were retained as purposively selected contrasting exemplars for subsequent instrumental characterization.

### 3.2. Artificial Saliva-Mediated Oral Disintegration

The artificial saliva had a pH of 6.8 ± 0.1 and an apparent viscosity of 2.73 ± 0.42 mPa·s at 10 s^−1^ and 25 °C. At 37 °C, friction coefficients were 0.1307 ± 0.013 and 0.0874 ± 0.012 at approximately 0.01 and 1000 mm/s, respectively, and the normalized log-velocity area under the friction curve was 0.2138 ± 0.036. Values are mean ± SD from three independently prepared saliva samples (*n* = 3). These measurements characterize the prepared fluid under the specified conditions and do not establish equivalence to human saliva.

**Figure 4:**
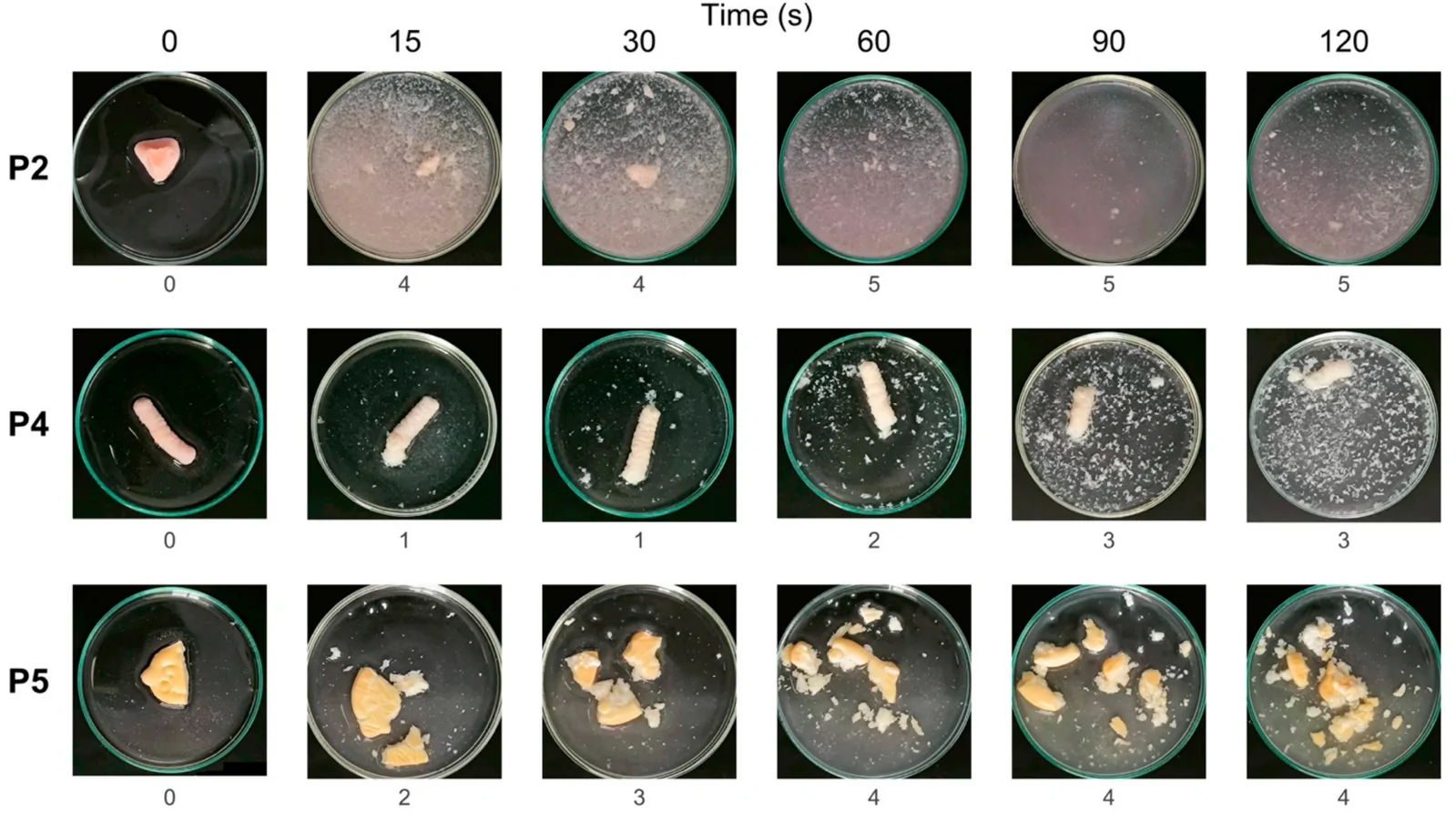
Representative photographic sequences of artificial saliva-mediated oral disintegration of P2, P4, and P5 over 0–120 s. Images show a representative specimen at each observation time (0, 15, 30, 60, 90, and 120 s); different specimens were assessed at each time point. Numbers below the images indicate visual disintegration scores (0 = intact; 5 = complete disintegration).

Artificial saliva-mediated oral disintegration produced distinct time-dependent responses among P2, P4, and P5. Representative photographic sequences showed rapid structural disruption and loss of discrete visible material in P2, progressive erosion and fragmentation in P4, and fragmentation with persistence of comparatively large discrete fragments in P5 (Figure 4).

**Figure 5:**
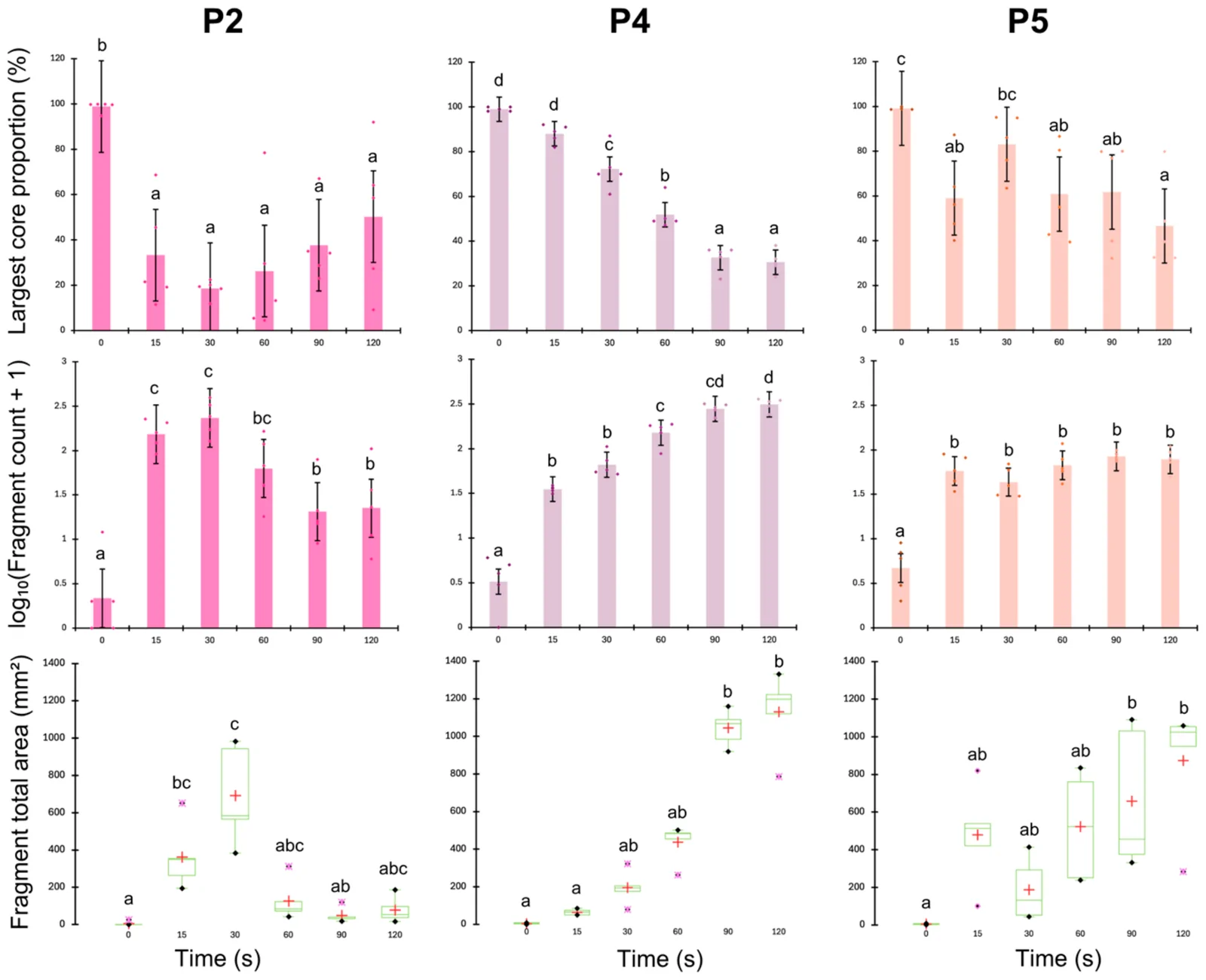
Time-dependent artificial saliva-mediated disintegration profiles of P2, P4, and P5. Largest core proportion is the projected area of the largest detected particle relative to total detected particle area, rather than retained food mass. Fragment count is presented as log_10_(fragment count + 1), and fragment total area excludes the largest detected particle. Five separate specimens were assessed once per product per time point (*n* = 5). Largest core proportion and transformed fragment count are shown as mean ± SD with individual observations; fragment total area is shown as box plots with individual observations. Dots represent individual observations. In the box plots, red plus signs (+) indicate means, and magenta crosses (×) mark outlying observations. Different letters indicate significant differences among time points within each product (*p* < 0.05).

These contrasting observed patterns were described using changes in largest core proportion, fragment count, and fragment total area over 120 s (Figure 5).

P2 underwent pronounced structural disruption during the early stage of the assay. The representative visual disintegration score increased from 0 at 0 s to 4 at 15–30 s and reached 5 from 60 s onward (Figure 4). Largest core proportion decreased sharply from 98.9% at 0 s to 33.3% at 15 s and 18.5% at 30 s (*p* < 0.0001); all saliva-exposed time points were significantly lower than the 0-s value, although substantial between-specimen variability was observed at later times (Figure 5). Fragment count also changed significantly with time (*p* < 0.0001), increasing rapidly during the early stage, reaching its highest transformed mean at 30 s, and subsequently declining. Fragment total area followed a similar temporal pattern (Kruskal–Wallis, *p* < 0.0001), with an early increase and maximum at 30 s followed by lower values during the later stages.

In contrast, P4 exhibited a progressive disintegration pattern throughout the assay. The representative visual score remained at 1 at 15–30 s, increased to 2 at 60 s, and reached 3 at 90–120 s (Figure 4). Largest core proportion progressively decreased from approximately 99% at 0 s to approximately 30% at 120 s, with significant separation among successive stages of disintegration (Figure 5). Concurrently, fragment count increased progressively (*p* < 0.0001), with the highest values observed at 90–120 s. Fragment total area showed a corresponding increase (Kruskal–Wallis, *p* < 0.0001), reaching approximately 1045 and 1132 mm^2^ at 90 and 120 s, respectively, with these later time points forming the highest significance group.

P5 showed a more variable and less monotonic response. The representative visual score increased from 0 at 0 s to 2 at 15 s, 3 at 30 s, and 4 from 60 to 120 s (Figure 4). Largest core proportion differed significantly with time (*p* = 0.001) but fluctuated among intermediate sampling points rather than decreasing progressively, with substantial overlap among Tukey groups (Figure 5). Fragment count increased sharply following saliva exposure (*p* < 0.0001), but all time points from 15 to 120 s belonged to the same Tukey group and were significantly higher than the 0-s value. Fragment total area also differed significantly over time (Kruskal–Wallis, *p* < 0.001) and, despite considerable within-time variability, was greatest during the later stages, with 90 and 120 s belonging to the highest Dunn group.

### 3.3. Dynamic Mechanical Behavior of Hydrated Boluses

**Figure 6:**
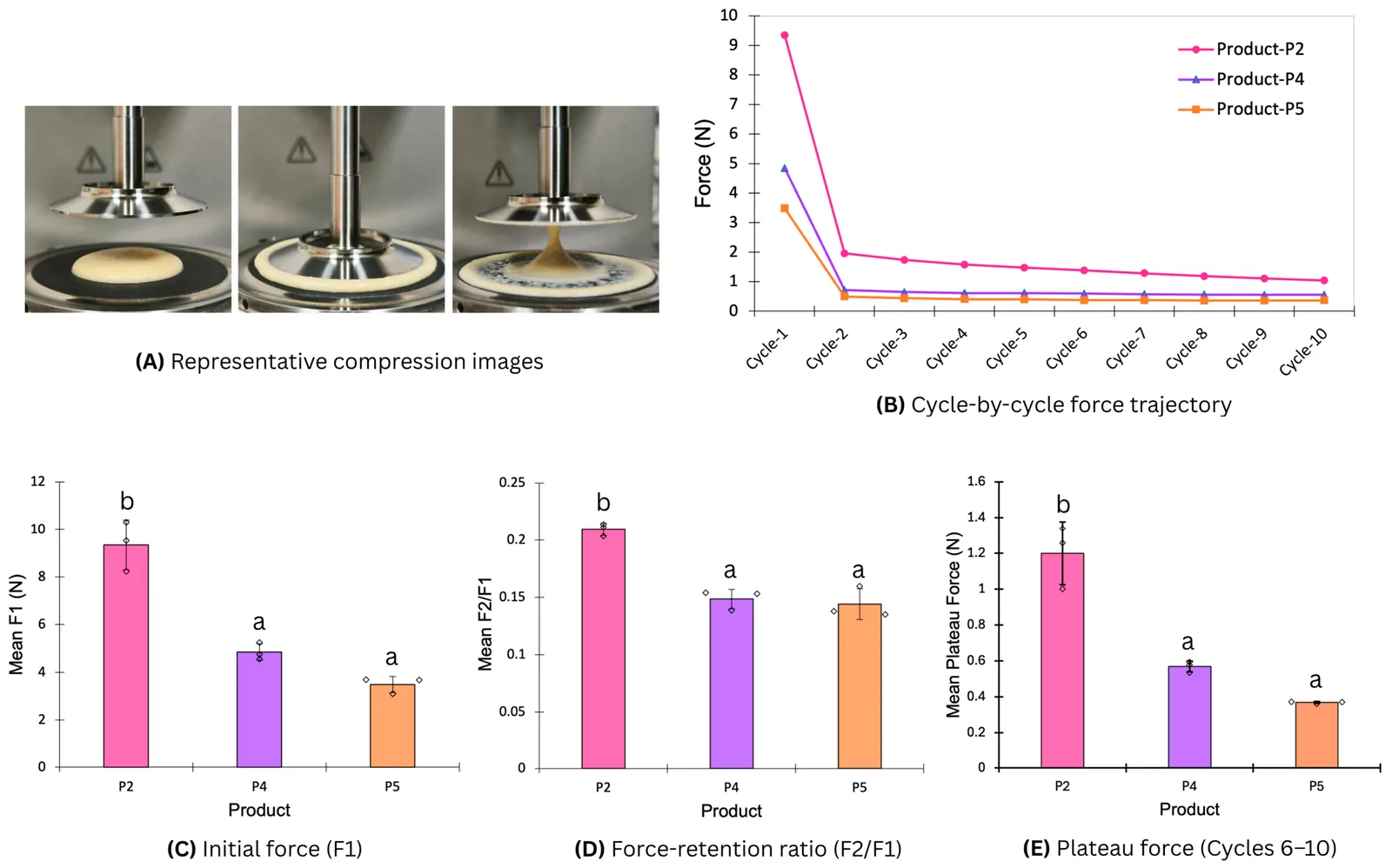
Dynamic instrumental mastication (DIM) characteristics of hydrated boluses prepared from P2, P4, and P5. Representative images obtained during repeated bolus compression are shown in (**A**), and cycle-by-cycle peak compression forces over ten consecutive cycles are shown in (**B**). Initial compression force (F1), force-retention ratio (F2/F1), and plateau force calculated as the mean peak compression force over Cycles 6–10 are presented in (**C**), (**D**), and (**E**), respectively. Data are presented as mean ± standard deviation of three separately prepared boluses per product (*n* = 3), with individual observations overlaid. The ten compression cycles were sequential measurements within each bolus preparation and did not constitute additional replicates. Different letters indicate significant differences among products (*p* < 0.05; Tukey’s HSD test).

Dynamic instrumental mastication (DIM) revealed distinct mechanical responses of the hydrated P2, P4, and P5 boluses during repeated compression (Figure 6). Representative images showed progressive deformation of the boluses across the compression–decompression sequence (Figure 6A). The cycle-by-cycle force trajectories showed a pronounced decrease in peak compression force after the first cycle, followed by smaller changes during subsequent cycles (Figure 6B). P2 maintained the highest force response throughout the ten cycles, P5 the lowest, and P4 showed an intermediate trajectory.

Initial compression force (F1) differed significantly among products (*p* < 0.05; Figure 6C). P2 exhibited the highest F1 (9.30 ± 1.08 N), followed by P4 (4.85 ± 0.36 N) and P5 (3.49 ± 0.35 N). P2 differed significantly from both P4 and P5, whereas P4 and P5 did not differ significantly.

The force-retention ratio (F2/F1) also differed significantly among products (*p* < 0.05; Figure 6D). P4 (0.148 ± 0.010) and P5 (0.144 ± 0.015) showed significantly lower ratios than P2 and did not differ significantly from each other.

Plateau force, calculated as the mean peak compression force over Cycles 6–10, differed significantly among products (*p* < 0.05; Figure 6E). P2 exhibited the highest plateau force (1.20 ± 0.18 N), followed by P4 (0.57 ± 0.03 N) and P5 (0.37 ± 0.01 N). As observed for F1 and F2/F1, P2 differed significantly from P4 and P5, whereas P4 and P5 did not differ significantly.

### 3.4. Rheological and Tribological Characteristics of Hydrated Boluses

**Figure 7:**
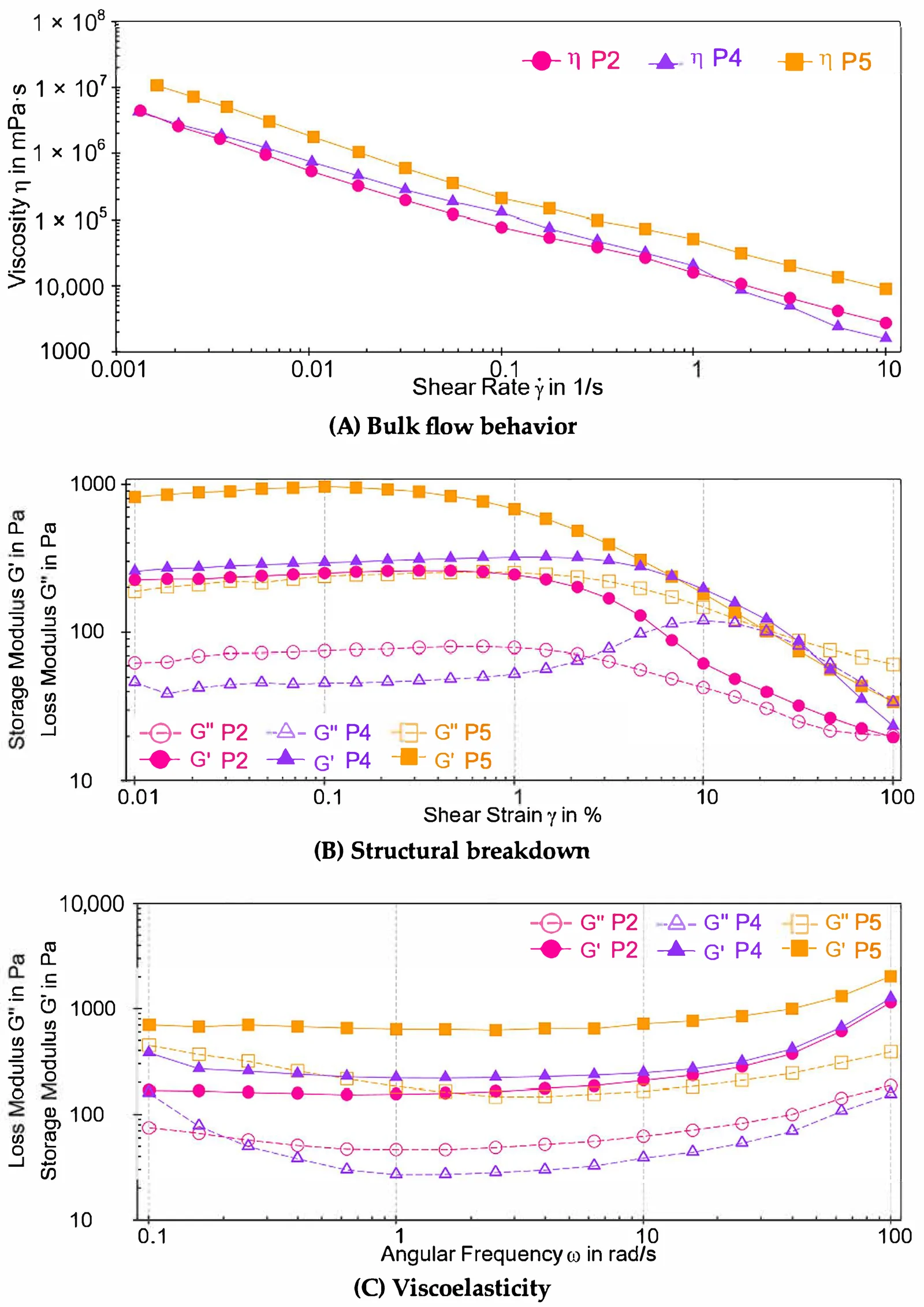

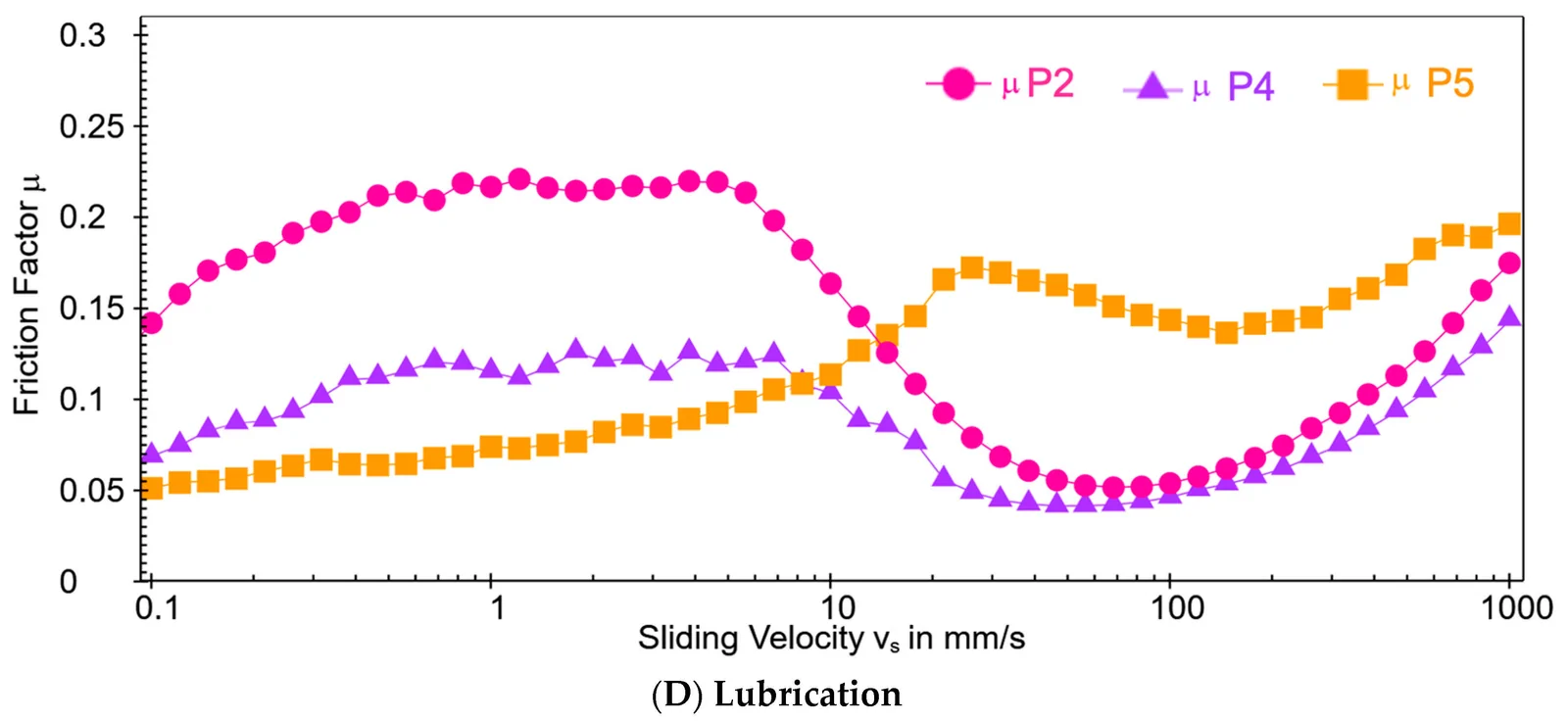
Rheological and tribological characterization of artificial saliva-hydrated boluses prepared from P2, P4, and P5. (**A**) Apparent viscosity as a function of shear rate during steady-shear measurements. (**B**) Storage modulus (*G*′) and loss modulus (*G*″) as a function of strain during oscillatory amplitude sweeps. (**C**) *G*′ and *G*″ as a function of angular frequency during oscillatory frequency sweeps. (**D**) Friction coefficient as a function of sliding velocity during Stribeck tribology measurements, shown over 0.1–1000 mm/s. P2, P4, and P5 are the selected exemplars assigned to the provisional protein-based cellular, expanded starch-based cellular, and dense biscuit categories, respectively. Measurements were performed using three separately prepared boluses per product for each measurement protocol (*n* = 3); points along each curve represent measurement conditions within a preparation, rather than additional replicates.

The prepared P2, P4, and P5 boluses differed across steady-shear, oscillatory, and tribological measurements (Table 3; Figure 7).

#### 3.4.1. Steady-Shear Flow Behavior

Apparent viscosity generally decreased with increasing shear rate for all three boluses (Figure 7A). Apparent viscosity at 1 s^−1^ (*η*_1_) followed the numerical order P5 > P4 > P2, although the product effect was not significant and P4 showed substantial between-preparation variability (Table 3).

Power-law fits over 0.1–10 s^−1^ yielded positive flow behavior indices for P2 (*n* = 0.28 ± 0.05) and P5 (*n* = 0.32 ± 0.01), consistent with shear-thinning behavior. Preparation-level log–log R^2^ values ranged from 0.921 to 0.975 for P2 and from 0.981 to 0.987 for P5. P5 exhibited a numerically higher consistency coefficient (*K* = 43.8 ± 2.0 Pa·s*^n^*) than P2 (15.9 ± 0.5 Pa·s*^n^*).

In contrast, P4 showed irregular or decreasing shear-stress responses over the fitting range, with inconsistent fitted exponents and R^2^ values of 0.055–0.703. Inspection of the fit residuals also indicated inadequate representation by a single power-law relationship. The negative fitted exponent was therefore treated as evidence of model inadequacy rather than a meaningful material property. P4’s fitted *K* and *n* were excluded from Table 3, and *K* and *n* were reported descriptively for P2 and P5 without inferential comparisons. P4 was characterized using its measured apparent-viscosity curve and *η*_1_.

#### 3.4.2. Oscillatory Amplitude-Sweep Behavior

Amplitude-sweep measurements showed changes in viscoelastic structure with increasing strain (Figure 7B). Storage modulus at 1% strain (*G*′_1%_) differed significantly among products (*p* = 0.003; Table 3). P5 exhibited the highest *G*′_1%_ (646.4 ± 38.2 Pa), significantly exceeding P2 and P4, which did not differ significantly from each other.

Critical strain (*γ*_c_) did not differ significantly among products (*p* = 0.482). Flow-point stress (*τ*_f_) differed among products (Kruskal–Wallis H = 7.20, Monte Carlo *p* = 0.003). Dunn’s comparisons with Holm–Bonferroni adjustment indicated higher *τ*_f_ for P4 (60.0 ± 31.4 Pa) than P2 (25.4 ± 1.1 Pa; adjusted *p* = 0.022). P5 (29.4 ± 1.2 Pa) did not differ significantly from either product.

#### 3.4.3. Oscillatory Frequency-Sweep Behavior

Frequency-sweep measurements further differentiated the viscoelastic characteristics of the three boluses (Figure 7C). At the reference angular frequency of 1 rad s^−1^, *G*′, *G*″, and tan *δ* differed significantly among products (*p* < 0.0001; Table 3). P5 exhibited the highest *G*′ (616.6 ± 21.0 Pa), followed by P4 (242.9 ± 23.5 Pa) and P2 (176.6 ± 32.9 Pa), with all three products differing significantly. For *G*″, P5 exhibited the highest value, P2 was intermediate, and P4 exhibited the lowest value.

At 1 rad s^−1^, tan *δ* was below unity for all three boluses and was significantly lower for P4 (0.131 ± 0.008) than for P2 (0.307 ± 0.013) and P5 (0.285 ± 0.001), which did not differ significantly from each other. Thus, *G*′ exceeded *G*″ for all three products at the selected reference frequency, with P4 exhibiting the lowest relative contribution of the viscous component.

#### 3.4.4. Tribological Behavior

Stribeck curves showed distinct frictional responses across sliding velocity (Figure 7D). Boundary friction coefficient (*μ*_B_) showed a significant overall product effect (*p* = 0.041), with mean values ranging from 0.028 to 0.100 (Table 3). P2 showed the highest numerical mean (0.100 ± 0.049), followed by P4 (0.033 ± 0.009) and P5 (0.028 ± 0.001); however, Tukey’s HSD test did not identify significant pairwise differences among products.

High-speed friction coefficient (*μ*_high_) also showed a significant overall product effect (*p* = 0.049), but no significant pairwise differences were detected by Tukey’s HSD test. P5 showed the highest numerical mean (0.191 ± 0.004), whereas P2 (0.161 ± 0.012) and P4 (0.162 ± 0.019) showed similar numerical values.

The normalized log-velocity area under the Stribeck curve (nAUC_log _*_v_*) provided clearer product differentiation (*p* = 0.005). P2 exhibited the highest nAUC_log _*_v_* (0.154 ± 0.028), significantly exceeding P4 (0.081 ± 0.003) and P5 (0.104 ± 0.009), whereas P4 and P5 did not differ significantly.

## 4. Discussion

### 4.1. Initial Structure and Mechanical–Acoustic Diversity of Commercial Snacks

The dry-state measurements indicate that hardness alone did not describe the fracture profiles of the seven sampled snacks. Mechanical and acoustic measurements capture complementary aspects of brittle-food failure, including fracture force, deformation, and acoustic events [8,23].

The dispersion of the four C2 products in PCA illustrates variation within the provisional matrix categories. Formulation, processing, geometry, and architecture may contribute jointly to fracture behavior; expanded-snack studies likewise associate mechanical and acoustic properties with expansion, porosity, density, and formulation [8,23,28]. Composition therefore provides explanatory context, but its individual contribution cannot be isolated in this commercial-product comparison.

The selected exemplars provided contrasts for examining hydration responses beyond dry-state fracture. Intact-food properties need not correspond directly to hydrated-material properties because breakdown, hydration, dilution, and particle interactions alter the material during oral processing [7,8,9,10].

### 4.2. Product-Specific Pathways of Artificial Saliva-Mediated Disintegration and Bolus Formation

The observed disintegration patterns differed among the three exemplars, indicating that their dry-state hardness ranking did not correspond consistently to their responses to artificial saliva. Hydration and food–saliva interactions can alter particle interactions and material properties during breakdown [8,9,18].

P2 combined relatively high dry-state fracture resistance with rapid disruption during artificial saliva exposure. Its apparent cellular architecture may contribute to this response, but architectural effects cannot be separated from composition, formulation, or processing. Studies of freeze-dried and dried dairy systems identify both structure and composition as relevant to rehydration [17,29,30]. The later decline in detected fragment count and area was compatible with dispersion, dissolution, aggregation, or loss below detection criteria; the images could not distinguish these contributions.

P4 showed progressive erosion and fragmentation and required the highest operational saliva-to-sample ratio to reach the cohesive-bolus endpoint. Fluid availability can influence bolus hydration and particle characteristics [31,32], but ASODA and bolus preparation were separate assays. Their results therefore describe complementary P4 responses rather than a continuous sequence linking disintegration to bolus formation.

Coarse particles remained detectable during P5 disintegration. Its apparent dense architecture and comparatively higher fat content provide possible explanatory context, but their contributions cannot be separated from formulation and processing. Biscuit mastication studies similarly describe particle fragmentation, saliva incorporation, and subsequent agglomeration during bolus formation [13].

The descriptors must be interpreted jointly: an increase in largest core proportion can reflect loss of smaller detectable particles or aggregation, rather than structural recovery. They describe detectable particulate material without establishing a mass balance or uniquely identifying breakdown mechanisms.

### 4.3. Whole-Bolus Mechanical, Rheological, and Tribological Differentiation

The contrasting DIM and rheological rankings show that resistance to repeated whole-bolus compression differed from resistance to shear and oscillatory deformation. P2 had the highest DIM forces, whereas P5 had the highest measured viscoelastic moduli and a numerically higher fitted *K* than P2; P4’s *K* was not reported. DIM and rotational rheology interrogate different loading modes, supporting their complementary use [7,19,20,21,22]. Although tongue–palate compression motivates instrumental compression testing [27], the measured forces describe the specified geometry and loading protocol rather than toddler eating effort.

The coexistence of persistent particles during P5 ASODA and high moduli in separately prepared boluses is compatible with a contribution from particulate material but does not demonstrate preservation of the original architecture. P4 showed the lowest tan δ at the reference frequency, indicating the strongest elastic dominance relative to its viscous response. Its irregular steady-shear response was inadequately described by the power-law model; poor fitting does not identify a specific material mechanism. Product-specific hydration ratios also limit separation of hydration effects from formulation and matrix characteristics [9,10,33].

P2’s higher integrated friction, despite comparatively low viscoelastic moduli, illustrates that bulk and interfacial measurements need not yield the same ranking. Friction depends on the material within the contact, surface interactions, and measurement configuration, as well as bulk properties [7,15,21,34,35,36]. The present measurements describe lubrication under defined instrumental conditions and do not directly measure oral comfort, oral transport, or swallowing ease. Sensory associations reported elsewhere require validation for the foods, conditions, and population studied [14].

### 4.4. Integrated Food-to-Bolus Transformation Pathways and Implications

Across the assays, the selected exemplars showed product-specific patterns whose rankings depended on the measurement mode. Here, ‘food-to-bolus transformation’ denotes dry-food fracture testing, artificial saliva-mediated disintegration, and characterization of separately prepared hydrated boluses. The findings are consistent with a possible contribution from matrix architecture but do not establish structural causality or a continuous sequence linking disintegration to bolus properties. Integrating the assays provides complementary descriptors beyond those obtainable from a single measurement, consistent with broader food oral-processing frameworks [7,8,9,24,25].

Comparison across assays identified associations between initial product characteristics and product-specific instrumental responses. These observations are consistent with the hypothesis that matrix architecture may contribute to transformation behavior but do not establish an independent causal effect of structure because architecture, composition, formulation, and processing history varied together. Product rankings also changed across measurement modes, indicating that dry-state fracture characteristics alone did not specify the measured hydrated-material responses. Among the three exemplars, the dry-state hardness ranking did not correspond consistently to the observed disintegration patterns, and greater bulk rheological strength did not necessarily correspond to greater friction. The multi-stage approach therefore revealed complementary dimensions of oral-processing behavior that would have been obscured by reliance on dry fracture, disintegration, rheology, or tribology alone. This interpretation is consistent with structure-based frameworks of food oral processing in which texture is understood through the sequence of physical and chemical transformations occurring during breakdown—including particle reduction, hydration, dissolution, surface interactions, and bolus formation—rather than from the intact food structure alone [24].

The framework may inform exploratory product comparison and hypotheses for product development. Relating these descriptors to developmental suitability, oral comfort, or swallowing requires validation against relevant feeding and oral-processing outcomes in the intended population [1,37].

The study screened seven products—two C1, four C2, and one C3—and examined three purposively selected exemplars in detail. Specimen and preparation replication did not increase the number of commercial products or establish lot independence. The provisional categories were not quantitatively validated, PCA was based on seven product-level observations, and contrast-based selection may accentuate differences. Findings therefore apply to the sampled material under the tested conditions. Structure, composition, geometry, formulation, and processing were confounded; testing structural causality requires controlled formulations with independently manipulated and quantified architecture, matched geometry, and controlled hydration.

Image analysis excluded undetectable material and could not distinguish dissolution, dispersion, aggregation, or fragmentation below its detection criteria. Saliva ratios defined an operational bolus endpoint rather than physiological saliva incorporation. The instrumental assays isolate selected transformations and contact conditions without reproducing dynamically changing saliva, compliant oral surfaces, muscular activity, or sensory feedback [7,18,19,35,38]. No toddler oral-processing or swallowing outcomes were measured, and physiological relevance remains unvalidated.

## 5. Conclusions

This exploratory study identified differences in mechanical–acoustic fracture behavior among seven sampled commercial toddler-oriented snacks and contrasting in vitro responses among three purposively selected exemplars. P2 showed rapid structural disruption, P4 showed progressive disintegration and a greater operational hydration requirement, and P5 showed fragmentation with persistence of coarse particulate material. Hydrated boluses also differed in their instrumental responses, with rankings varying across whole-bolus compression, rheology, viscoelasticity, and tribology.

These findings suggest associations between initial product characteristics and subsequent instrumental responses under the tested conditions. They apply to the sampled commercial material and do not establish class-wide transformation pathways or causal effects of food architecture. Controlled-formulation studies that independently manipulate and quantify structural variables are needed to test their contributions.

The multi-stage approach provides complementary instrumental descriptors for comparing commercial toddler-oriented finger foods. Validation against oral-processing and feeding outcomes in the target population is required before using these descriptors to infer oral comfort, ease of eating, swallowing performance, or developmental suitability.

## Figures and Tables

**Table 1:** AED-3PB mechanical–acoustic fracture characteristics of seven commercial toddler-oriented snack products.

Product	Hardness (g)	Fracturability (mm)	Count_Peaks	Jagged_Smooth_Ratio
P1	531.6 ± 102.0	6.66 ± 0.55 ^bc^	1604 ± 56 ^ab^	823.2 ± 39.9 ^c^
P2	2717.9 ± 896.5	7.00 ± 1.04 ^c^	1675 ± 41 ^b^	870.3 ± 20.6 ^cd^
P3	3426.3 ± 774.7	3.62 ± 0.55 ^a^	1542 ± 89 ^ab^	622.0 ± 49.0 ^a^
P4	1567.4 ± 125.1	6.66 ± 0.47 ^bc^	1500 ± 41 ^a^	827.3 ± 21.0 ^cd^
P5	2710.2 ± 277.0	5.38 ± 0.41 ^b^	1602 ± 116 ^ab^	873.9 ± 21.5 ^cd^
P6	1762.6 ± 339.6	6.76 ± 0.86 ^bc^	1616 ± 68 ^ab^	885.2 ± 19.2 ^d^
P7	1879.5 ± 304.3	2.63 ± 0.84 ^a^	1512 ± 44 ^a^	745.5 ± 45.9 ^b^
Product effect	Welch *p* < 0.0001	ANOVA *p* < 0.0001	ANOVA *p* = 0.003	ANOVA *p* < 0.0001
*η* ^2^	0.834	0.861	0.461	0.873

Values are mean ± SD of separately tested snack specimens (*n* = 4–8 per product); each specimen was tested once. For fracturability and the acoustic descriptors, different lowercase superscript letters indicate significant differences according to Tukey’s HSD (*p* < 0.05). Hardness was evaluated using Welch’s ANOVA because variances were unequal; no pairwise significance groups are reported for hardness. Count_Peaks represents the number of positive acoustic peaks exceeding the specified threshold, and Jagged_Smooth_Ratio represents the ratio of the linear distance of the original acoustic trace to that of its smoothed trace, expressed as a percentage.

**Table 2:** Loadings and contributions of AED-3PB variables to the first two principal components.

Variable	PC1 Loading	PC1 Contribution (%)	PC2 Loading	PC2 Contribution (%)
Hardness	−0.467	9.03	0.846	66.62
Fracturability	0.918	34.84	−0.008	0.01
Count_Peaks	0.722	21.56	0.598	33.28
Jagged_Smooth_Ratio	0.915	34.58	−0.032	0.09
Variance explained (%)	60.50	—	26.88	—
Cumulative variance (%)	60.50	—	87.38	—

**Table 3:** Rheological and tribological characteristics of artificial saliva-hydrated boluses prepared from P2, P4, and P5.

Parameter	Unit	P2	P4	P5
**Steady-shear flow**				
Consistency coefficient, *K*	Pa·s*^n^*	15.9 ± 0.5	—	43.8 ± 2.0
Flow behavior index, *n*	-	0.28 ± 0.05	—	0.32 ± 0.01
Apparent viscosity at 1 s^−1^, *η*_1_	Pa·s	18.0 ± 3.0	40.7 ± 34.1	49.7 ± 1.8
**Oscillatory amplitude sweep**				
Storage modulus at 1% strain, *G*′_1%_	Pa	285.7 ± 44.3 ^a^	425.9 ± 112.8 ^a^	646.4 ± 38.2 ^b^
Critical strain, *γ*_c_	%	4.70 ± 1.43	6.63 ± 5.69	3.08 ± 0.24
Flow-point stress, *τ*_f_	Pa	25.4 ± 1.1 ^a^	60.0 ± 31.4 ^b^	29.4 ± 1.2 ^ab^
**Oscillatory frequency sweep**				
*G*′ at 1 rad/s	Pa	176.6 ± 32.9 ^a^	242.9 ± 23.5 ^b^	616.6 ± 21.0 ^c^
*G*″ at 1 rad/s	Pa	54.5 ± 12.7 ^b^	31.9 ± 4.8 ^a^	176.0 ± 6.7 ^c^
tan *δ* at 1 rad/s	-	0.307 ± 0.013 ^b^	0.131 ± 0.008 ^a^	0.285 ± 0.001 ^b^
**Stribeck tribology**				
Boundary friction coefficient, *μ*_B_	-	0.100 ± 0.049 ^a^	0.033 ± 0.009 ^a^	0.028 ± 0.001 ^a^
High-speed friction coefficient, *μ*_high_	-	0.161 ± 0.012 ^a^	0.162 ± 0.019 ^a^	0.191 ± 0.004 ^a^
Normalized log-velocity AUC, nAUC_log _*_v_*	-	0.154 ± 0.028 ^b^	0.081 ± 0.003 ^a^	0.104 ± 0.009 ^a^

Values are mean ± SD of three separately prepared boluses per product for each measurement protocol (*n* = 3). Within each row, values sharing a lowercase superscript letter do not differ significantly. Pairwise comparisons used Tukey’s HSD, except for flow-point stress (*τ*_f_), which used Dunn’s test with Holm–Bonferroni adjustment (*p* < 0.05). *K* and *n* are reported descriptively for P2 and P5 without inferential comparisons. ‘—’ indicates that these parameters were not reported for P4 because the power-law model inadequately described its measured response.

## Data Availability

The data supporting the findings of this study are provided in the article and its Supplementary Materials. Additional raw instrumental data are available from the corresponding author upon reasonable request.

## Supplementary Materials

The following supporting information can be downloaded at: https://www.mdpi.com/article/10.3390/foods15213771/s1, Table S1: Commercial product information, provisional matrix categories, and measured proximate composition of the sampled toddler-oriented snacks; Dataset S1: Minimal dataset supporting the mechanical–acoustic, artificial saliva-mediated oral disintegration, dynamic instrumental mastication, rheological, and tribological analyses.
